# Supplementary figures and images for: Association of primary postpartum hemorrhage with inter-pregnancy interval in urban South Ethiopia: A matched nested case-control study
Source: PLoS One. 2022 Jul 18;17(7):e0271216. doi: 10.1371/journal.pone.0271216 (PMC9292068; doi:10.1371/journal.pone.0271216)

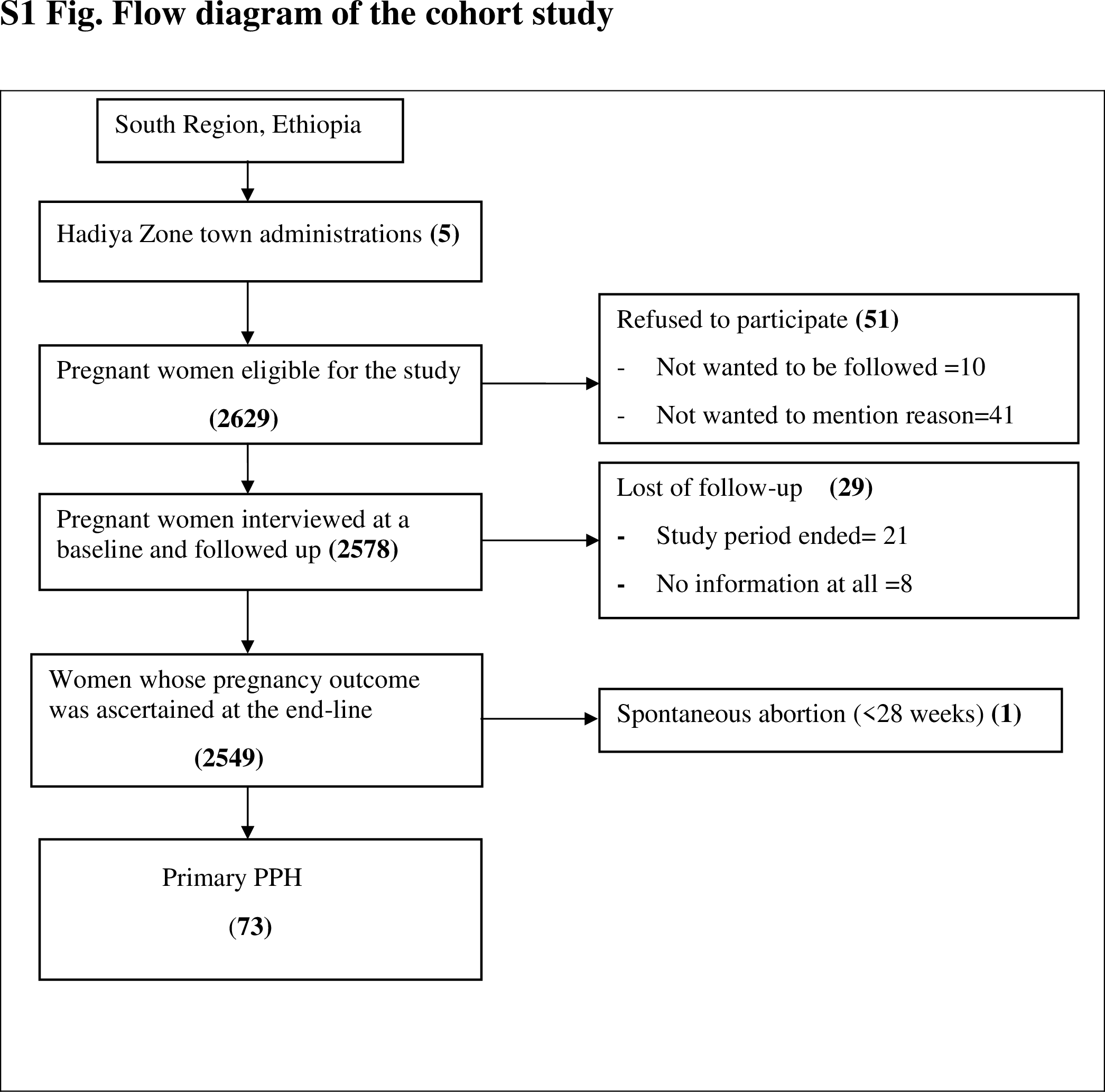

Supplement: S1 Fig — (TIF) [file pone.0271216.s001.tif]
