# Supplementary material for: Association of primary postpartum hemorrhage with inter-pregnancy interval in urban South Ethiopia: A matched nested case-control study
Source: PLoS One. 2022 Jul 18;17(7):e0271216. doi: 10.1371/journal.pone.0271216 (PMC9292068; doi:10.1371/journal.pone.0271216)
Supplement: S1 File — (DOCX) [file pone.0271216.s002.docx]

**S1 File. Measures taken against potential sources of bias**

In this study, bias might be introduced while estimating blood loss, selection of participants during initial enrollment for the main cohort and data collection, and could be attempted to minimize by early recognizing potential sources during design stage and analysis.

Selection bias: In our study, determining gestational age by Ultrasound was not feasible for all study participants and also it was not available in most of health facilities in Ethiopia. Because of these reasons determining gestational age by LMP was an alternative option. On the other side, excluding women who did not remember LMP leads to selection bias. Thus, for those women who couldn’t be able to recall LMP due to breast feeding, contraceptive method use, and other reasons, we used Ultrasound as a solution to minimize selection bias. For this study cases were individually matched for age group and location, and the controls were randomly selected from risk set (data frame). This might help in reducing bias in this study. We included all pregnant women during the nine months enrollment so that this population level study also helps to minimize selection bias. Due to cost constraints for laboratory tests, enrolment was made after first trimester because first trimester pregnancy was detected mainly via laboratory tests and some women might not know whether they are pregnant (another potential source of selection bias). Hence we enrolled pregnant women who had already confirmed pregnancy or visible pregnancy.

Recall bias: happened when the participants asked the date of preceding child birth and last menstrual period. In urban community, it is customary to see birth date ceremonies, it was used in addition to verbal report. Immunization card was also used where available which help to see the date that immunization was initiated, usually initiated at 45 days of delivery in addition to those vaccines given at birth. Family members such as husband, mother in-law and others who exactly remember date of recent child birth were used to support the women in recalling. Furthermore, we limited time of the most recent child birth date, within the last five years from the date of data collection to minimize bias related to recalling.

Misclassification bias: might happen when some subjects having a primary PPH remain undiagnosed or some subjects might be diagnosed for PPH with minimal bleeding that occurs naturally since there is no clear-cut and accurate means to estimate blood loss in the study settings. Thus, cases and controls likely to be misclassified. This bias was attempted to minimize by consulting gynecologists and emergency medicine specialists in addition to those midwives who had a training on basic and comprehensive emergency obstetric care services.

Bias due to sparse data: occurs when sample size is small. We used a case to control ratio of 1:4 to increase sample size and precision. We used matched analysis (conditional logistic regression) which was developed as a remedy for the sparse data bias and it is a standard for analyzing matched case control data.
