# Supplementary material for: Association of primary postpartum hemorrhage with inter-pregnancy interval in urban South Ethiopia: A matched nested case-control study
Source: PLoS One. 2022 Jul 18;17(7):e0271216. doi: 10.1371/journal.pone.0271216 (PMC9292068; doi:10.1371/journal.pone.0271216)
Supplement: S1 Table — (DOC) [file pone.0271216.s004.doc]

**S1 Table. Sensitivity analysis using full cohort data, unconditional logistic regression**

| **Variables** | **Primary PPH** | | **Crude OR (95%CI)** | **Adjusted OR (95%CI)** | **AF (95% CI)** |
| --- | --- | --- | --- | --- | --- |
|  | **Yes** | **No** |  |  |  |
| **Inter-pregnancy interval in months** | | | | | |
| <24 | 54(74) | 1203(48.6) | 3.01(1.8, 5.1)^***^ | 3.26(1.9, 5.7)^***^ | 69.3% (46.2, 82.4) |
| 24-60 | 19(26) | 1272(51.4) | 1 | 1 | 1 |
| **Antepartum hemorrhage** | | | | | |
| Absent | 62(84.9) | 2421(97.8) | 1 | 1 | 1 |
| Present | 11(15.1) | 54(2.2) | 7.95(3.9, 15.9)^***^ | 6.50(2.9, 14.1)^***^ | 84.6%(66.6, 92.9) |
| **Mode of delivery** | | | | | |
| Spontaneous vaginally | 62(84.9) | 2272(91.8) | 1 | 1 |  |
| Cesarean section | 4(5.5) | 110(4.4) | 1.33(0.48, 3.7) | 0.47(0.1, 1.5) |  |
| Instrumental | 7(9.6) | 93(3.8) | 2.76(1.2, 6.2)^*^ | 1.04(0.39, 2.8) |  |
| **Antenatal care visits** | | | | | |
| 0-3 | 40(54.8) | 1740(70.4) | 1 | 1 | 1 |
| ≥ 4 | 33(45.2) | 731(29.6) | 1.96(1.2, 3.1)^**^ | 1.92(1.2, 3.1)^*^ | 47.9%(13.8, 68.1) |
| **Pre-eclampsia** | | | | | |
| Absent | 70(95.9) | 2436(98.4) | 1 | 1 |  |
| Present | 3(4.1) | 39(1.6) | 2.68(0.81, 8.9)⋅ | 2.83(0.78, 10.3) |  |
| **Prolonged labour** | | | | | |
| Absent | 53(72.6) | 2314(93.5) | 1 | 1 | 1 |
| Present | 20(27.4) | 161(6.5) | 5.42(3.2, 9.3)^***^ | 4.90(2.6, 9.3)^***^ | 79.6%(61.1, 89.3) |
| **Mal-presentation** | | | | | |
| Absent | 69(94.5) | 2399(96.9) | 1 | 1 |  |
| Present | 4(5.5) | 76(3.1) | 1.83(0.65, 5.2)^⋅^ | 1.14(0.34, 3.7) |  |
| **Type of pregnancy** | | | | | |
| Singleton | 65(89) | 2441(98.6) | 1 | 1 | 1 |
| Multiple | 8(11) | 34(1.4) | 8.84(3.9, 19.8)^***^ | 10.07(4.1, 24.7)^***^ | 90.1%(75.6, 95.9) |
| **Birth weight** | | | | | |
| ≤ 4000 gm | 60(82.2) | 2283(92.2) | 1 | 1 | 1 |
| >4000 gm | 13(17.8) | 192(7.8) | 2.58(1.4, 4.8)^**^ | 2.28(1.1, 4.6)^*^ | 56.1%(12.3, 78.1) |
| **Parity** | | | | | |
| 1-2 | 50(68.5) | 1679(68) | 1 | 1 | 1 |
| 3-4 | 13(17.8) | 589(23.8) | 0.74(0.40, 1.4) | 0.89(0.46, 1.7) | -- |
| ≥ 5 | 10(13.7) | 202(8.2) | 1.66(0.8, 3.3)^⋅^ | 2.37(1.1, 5.0)^*^ | 57.8%(10.7, 8) |
| **Maternal age** | **--** | **--** | 1.07(1.0, 1.2)^*^ | 1.08(1.01, 1.2)^*^ | 7.4%(1.0, 15.9) |

Keys: Significant *** = P<0.001, ** = P<320 0.01, * = P<0.05. ▪ = P<0.25 OR: Odds Ratio. CI: Confidence Interval. 1 = reference category.
