# Supplementary material for: Association of primary postpartum hemorrhage with inter-pregnancy interval in urban South Ethiopia: A matched nested case-control study
Source: PLoS One. 2022 Jul 18;17(7):e0271216. doi: 10.1371/journal.pone.0271216 (PMC9292068; doi:10.1371/journal.pone.0271216)
Supplement: Attachment — Submitted filename: Response to Reviewers.docx [file pone.0271216.s005.docx]

**To:** **PLOS ONE** **Journal, Editorial Office**

**Subject:** Submitting a revised version of manuscript and response to **Editor and** **Reviewers.**

Ref: Submission ID PONE-D-21-27673

**Title of Article:** " Association of primary postpartum hemorrhage with inter-pregnancy interval in urban South Ethiopia: a matched nested case control study"

**Authors:**

Belayneh Hamdela Jena, Gashaw Andargie Biks, Yigzaw Kebede Gete, Kassahun Alemu Gelaye

We would like to thank the Editor for facilitating, commenting and giving the opportunity to revise our manuscript. We are also grateful to reviewers for sharing their views and constructive comments. The comments are very important which will improve the quality of our manuscript. The point-by-point responses for each of the comments and the revised manuscript are provided in the attached documents.

**Regards,**

**The authors!**

**Editor’s comments:**

Journal Requirements:

When submitting your revision, we need you to address these additional requirements.

1. Please ensure that your manuscript meets PLOS ONE's style requirements, including those for file naming.

**Authors’ response**: Dear editor, thank you again for your time and contribution. We looked at the PLOS ONE style requirements to edit the whole manuscript and revised accordingly.

2. We note that the grant information you provided in the ‘Funding Information’ and ‘Financial Disclosure’ sections do not match. When you resubmit, please ensure that you provide the correct grant numbers for the awards you received for your study in the ‘Funding Information’ section.

**Authors’ response:** Thank you dear editor, we edited the ‘funding information’.

3. Thank you for stating the following in the Acknowledgments Section of your manuscript: “We would like to thank the University of Gondar and Wachemo University for their financial support. We are very much thankful to study participants, data collectors, supervisors and health workers for their contributions.”

We note that you have provided funding information that is not currently declared in your Funding Statement. However, funding information should not appear in the Acknowledgments section or other areas of your manuscript. We will only publish funding information present in the Funding Statement section of the online submission form.

Please remove any funding-related text from the manuscript and let us know how you would like to update your Funding Statement. Currently, your Funding Statement reads as follows:

“The funders had no role in study design, data collection and analysis, decision to publish, or preparation of the manuscript.”

Please include your amended statements within your cover letter; we will change the online submission form on your behalf.

**Authors’ response:** Dear editor**,** we have revised the financial disclosure statements. We revised as “The authors received no specific funding for this work.”

In addition, we removed funding-related text from the manuscript.

4. We note that you have indicated that data from this study are available upon request. PLOS only allows data to be available upon request if there are legal or ethical restrictions on sharing data publicly. For more information on unacceptable data access restrictions, please see <http://journals.plos.org/plosone/s/data-availability#loc-unacceptable-data-access-restrictions>.

In your revised cover letter, please address the following prompts:

a) If there are ethical or legal restrictions on sharing a de-identified data set, please explain them in detail (e.g., data contain potentially sensitive information, data are owned by a third-party organization, etc.) and who has imposed them (e.g., an ethics committee). Please also provide contact information for a data access committee, ethics committee, or other institutional body to which data requests may be sent.

b) If there are no restrictions, please upload the minimal anonymized data set necessary to replicate your study findings as either Supporting Information files or to a stable, public repository and provide us with the relevant URLs, DOIs, or accession numbers. For a list of acceptable repositories, please see <http://journals.plos.org/plosone/s/data-availability#loc-recommended-repositories>.

We will update your Data Availability statement on your behalf to reflect the information you provide.

**Authors’ response:** Dear editor, we have uploaded dataset as supporting information (**S1 Dataset**).

5. We note that you have stated that you will provide repository information for your data at acceptance. Should your manuscript be accepted for publication, we will hold it until you provide the relevant accession numbers or DOIs necessary to access your data. If you wish to make changes to your Data Availability statement, please describe these changes in your cover letter and we will update your Data Availability statement to reflect the information you provide.

**Authors’ response:** Thank you dear editor, we provided data set as supporting information **(S1 Dataset).** We stated in online system as “All relevant data are within the manuscript and its Supporting Information files”.

6. Your ethics statement should only appear in the Methods section of your manuscript. If your ethics statement is written in any section besides the Methods, please delete it from any other section.

**Authors’ response:** Dear editor, thank you we put ethics statement in the methods section.

**Additional Editor Comments:**

This is an interesting study to assess the association between inter-pregnancy interval (IPI) and postpartum haemorrhage (PPH). The authors used a nested matched case-control in a cohort of pregnant recruited after the first trimester in a rural community. They provide a good reason for such an approach (improve statistical efficiency). Usually, this design as well allows the investigators to put extra resources (usually few) in a subsample (in this case use them in 365 rather than in the whole 2546).

Issues:
1. The background is good. However, we still lack a clear description of the potential mechanism the IPI would lead to PPH. And please, provide us with the concept of “primary PPH”.
**Authors’ response:** Thank you dear editor for your time and contribution to this manuscript. Your inputs will improve the quality of our manuscript. We acknowledge your comments. In the revised manuscript we added 1 paragraph about potential causal mechanisms. As we defined early in the paragraph, and subsequent paragraphs of the introduction section, primary PPH is excess bleeding that occurs within 24 hours after delivery. Primary refers to the time (within 24 hours). It is commonly due to direct causes such as uterine atony, retained placental tissues, uterine rupture/lacerations, etc, and the main cause of maternal death, as we reported in the introduction. If bleeding occurs after 24 hours but before 12 weeks after delivery, then it is defined as secondary PPH. Secondary PPH is usually secondary to puerperal sepsis/infection and other causes. Our focus was on primary due to its impact and feasibility of data on the diagnosis.
2. Please put citations for the software used including open-Epi, Epidata, Stata and R. And please write Stata not STATA.
**Authors’ response:** Dear editor, thank you! We put citations for each software inside the text of the revised manuscript, and we showed in the highlighted-text. Note: when removing post-hoc power analysis we also removed Stata, as you commented on comment number 6 below.
3. Why line 120 says cohorts?
**Authors’ response:** Thank you dear editor, it was editorial problem. Now we corrected it, as ‘a cohort’.
4. Lines 134 and 135 are critical as they define the controls. Please clarify, does this mean only women delivering without PPH; or women still at observation without the outcome?
**Authors’ response:** Thank you dear editor. We mean that ‘women delivering without PPH’. It did not include those who were lost of follow-up, and abortion cases. We corrected it in the text.
5. Correct the title. It should be “case-control” not “case control”.
**Authors’ response:** Thank you dear editor, we corrected as “case-control” throughout the manuscript.
6. The Power/Sample details suggest being a post-hoc power calculation. It is quite strange the authors would know in advance that the OR would be 2.97. Please clarify. If it is a post-hoc power please remove from the text.
- Please a similar concerns by the reviewer below.
**Authors’ response:** Thank you dear editor for the comment. We acknowledge your view. It was pot-hoc power, which was just to estimate whether the sample size was adequate or not. Now we removed it.
7. Line 71 - please correct. Africa and Asia are not countries. Are continents.
**Authors’ response:** thank you dear editor, we corrected as ‘continents’.
8. The bracket opened at the beginning of the line 83 never closes. Please check.
**Authors’ response:** Dear editor, we checked it, and closed the bracket.
9. Tables 1 and 2 please make sure all percentages have one decimal place.
**Authors’ response:** Dear editor, we checked for it, and all have only one decimal, except P-values in Table 1 and 2. Thank you!
10. In the limitations. Please move the sensitivity analysis to somewhere in the results.

**Authors’ response:** Thank you dear editor, now we moved the sensitivity analysis in the result section under the regression table. Just before, discussion section. Thank you for the input.

**Reviewers’ comments**

**Reviewer #1**

This manuscript reports a nested case control study looking at the association between interpregnancy interval (IPI) and the risk of postpartum haemorrhage (PPH) in Ethiopia and found that short IPI (<24 months) was associated with an increased chance of primary PPH. The data collection, study design and analysis are appropriate and the paper is reported clearly. I have a few queries and suggestions below:

1. It is not clear why a nested case control approach was used. If a cohort of 2456 pregnant women were recruited and classified on the basis of their most recent IPI, why was a cohort study not undertaken?

**Authors’ response:** Thank dear reviewer for your time and contribution to this manuscript. Your comments will improve the quality of our manuscript. We acknowledge your views. We considered a nested case-control study for the following reasons: we faced the outcome (primary PPH) was rare (for statistical purpose). In this case, case-control study is efficient (for rare case). We want to apply more robust statistical methods (matched and paired analysis). We did a matched analysis to increase the strength of analysis for more valid estimate. Matching for known confounder (e.g. age) in addition to adjustment for other risk factors in multivariable model increase statistical efficiency. Matching for a variable in the whole cohort is not feasible. In addition, matching for ‘location (kebele)’ makes some unobserved sources of variation more similar such as neighborhood and health service related characteristics. Kindly, see additional reasons in your comment number 4 below!

2. It seems a little odd that exactly half the cohort had a short IPI. Was this deliberately done at the time of recruitment?

**Authors’ response:** Thank you dear reviewer for this crucial comment. At the beginning of the study, our thinking was just to take equal number of exposed and unexposed groups. While completing the enrollment, we randomly excluded 32 individuals (as we showed in Sl flow diagram in previous submission) from the unexposed group just to equalize the sample size for both group. A similar comment as you was given by other reviewer in one of our study related to this (<https://doi.org/10.1186/s12884-021-04325-z> ), and the reviewer suggest us to go back to collect the outcomes status of those 32 individuals data dropped. Later, we the authors discussed, and decided to include the outcome data of the 32 participant by going back to the health facilities since we already had a baseline data at hand together with their addresses. So we trace back and collected the outcome data to include in the analysis. Thereby we also collected the data for the primary PPH too. Therefore, in this revised manuscript we added the information about those 32 participants in the sample size, flow diagram and figure as well. Of course, we could not get a woman diagnosed for primary PPH among those 32 participants. We also checked if they should be matched for age and location (kebele). However, the data of the 32 participants were not eligible for the matching and stratification. Thus, we did not include them in the analysis of the nested case-control study. Rather we report in sample size sections, figure and flow diagram in the revised manuscript. Dear reviewer as you really commented, and the same comment from the other reviewer in related article, it is not mandatory to take equal number of exposed and unexposed group for cohort study. Thank you again for your input.

3. Table 3 should present the variables adjusted for as the adjusted odds ratio.

**Authors’ response:** Thank you dear reviewer, we revised Table 3. Kindly, see the table 3 in the text in the revised manuscript.

4. "Despite the limitations, nested case control studies are efficient in elucidating temporal relationships, especially when supplemented with matching and stratification (conditional analysis)". This sentence in the discussion should be rephrased. I am not sure that this really qualifies as a nested case control study as the approach was that of a matched case control. Why is matching and stratification superior to adjustment - which has also been done in this analysis?

**Authors’ response:** Thank you dear reviewer, we removed it from the discussion as it was repeatedly used. Of course, we applied both matching & stratification and adjustment as well. Both matching and adjustment are used to control confounding. We are not making matching and stratification superior to adjustment. We matched for “age” as it is a well-known biological confounder in many of health-related outcomes including PPH. Matching for location makes cases and controls more similar in terms of neighborhood circumstances, so that the cases and controls represent the population from where they come from. For sparse data, data from different geographic location, and rare conditions, nested case-control study via applying conditional analysis (matched and paired analysis) is appropriate. Using both matching and adjustment increase statistical strength.

END!
