# Supplementary material for: Association of primary postpartum hemorrhage with inter-pregnancy interval in urban South Ethiopia: A matched nested case-control study
Source: PLoS One. 2022 Jul 18;17(7):e0271216. doi: 10.1371/journal.pone.0271216 (PMC9292068; doi:10.1371/journal.pone.0271216)
Supplement: Attachment — Submitted filename: Response to Reviewers.docx [file pone.0271216.s006.docx]

**To:** **PLOS ONE** **Journal, Editorial Office**

**Subject:** Submitting a revised version of manuscript and response to **Editor and** **Reviewers.**

Ref: Submission ID PONE-D-21-27673R1

**Title of Article:** " Association of primary postpartum hemorrhage with inter-pregnancy interval in urban South Ethiopia: a matched nested case control study"

**Authors:**

Belayneh Hamdela Jena, Gashaw Andargie Biks, Yigzaw Kebede Gete, Kassahun Alemu Gelaye

We would like to thank the Editor for facilitating, commenting and giving the opportunity to revise our manuscript. We are also grateful to reviewers for sharing their views and constructive comments. The comments are very important which will improve the quality of our manuscript. The point-by-point responses for each of the comments and the revised manuscript are provided in the attached documents.

**Regards,**

**The authors!**

**Editor’s comments:**

Journal Requirements:

Please review your reference list to ensure that it is complete and correct. If you have cited papers that have been retracted, please include the rationale for doing so in the manuscript text, or remove these references and replace them with relevant current references. Any changes to the reference list should be mentioned in the rebuttal letter that accompanies your revised manuscript. If you need to cite a retracted article, indicate the article’s retracted status in the References list and also include a citation and full reference for the retraction notice.

**Authors’ response**: Thank you dear editor for your time, immense contribution, and giving the opportunity to revise the manuscript. We acknowledge your constructive comments, and suggestions, which will improve the quality of our manuscript indeed. We have checked the references for any retraction, and no retracted references found.

Additional Editor Comments (if provided):

Thank you for responding to all our comments and questions.
The nested case-control design is not yet a commonly known design. This design is born within a cohort from which cases are identified. Then matched controls are found from the cohort by a density sampling (your case here) or baseline sampling.
The main advantage is that extra (and usually expensive measurements like, for example, biomarkers) information can be collected for this relatively small sample. In particular, the density sampling in a case-control can help approximate a relative-risk without the need for the low risk/incidence condition in the overall population. However, the case-control still potentially suffers from selection bias and we have to agree with the reviewer there is a lot calculations involved to analyse this.
Therefore, I would suggest:
1) Please, do the extra analysis (if data is available as the reviewer recommends)

**Authors’ response**: Thank you dear editor, we acknowledge your suggestion, and reviewer’s as well. We already have full data for the participants. Primary postpartum hemorrhage (PPH) was one of the outcomes hypothesized to have association with inter-pregnancy interval during designing the study. Thus, we have conducted the sensitivity analysis using 2548 sample data for which the outcome was ascertained, as suggested by reviewer#1, using logistic regression. The result of the sensitivity analysis (full data containing the regression analysis) was presented in supplementary table 1 (S1 Table).

We have included the result of the sensitivity analysis in the result section of the revised manuscript, under sensitivity analysis section, and supplementary table was also cited.

2) Comment about those results and put those tables in the Supplementary materials.

**Authors’ response:** Dear editor, as you can see from the supplementary table 1, no significant difference on the conclusion and the direction of association was observed between the two approaches (nested case-control study and cohort study), except very few difference on the estimates; that means AOR=2.97~3.0, 95% CI (1.6, 5.7) for the nested case-control study while AOR=3.26 ~3.3, 95% CI (1.9, 5.7) for the cohort study. In both study designs, the results are nearly the same, and the interpretation is the same: the odds of primary postpartum hemorrhage was 3 times higher for women with inter-pregnancy interval under 24 months as compared to women with inter-pregnancy interval 24-60 months. Since the data on primary postpartum hemorrhage was rare, and sparse because the primary PPH was from five urban settings, which are geographically diverse. For such sparse data, nested case-controls studies highly appropriate. Nested case-controls study is efficient with matching for known biological confounders such as age. Such matching, when needed, is not feasible for large sample of cohort, like ours 2548, when the outcome is rare or sparse. As you really mentioned above, nested case-control study is primarily relevant for rare conditions like biomarkers, it is also commonly being applicable for disease, death and behavioral conditions. Kindly, I have included just few literature (of course many) on nested case-control studies regarding the areas of application.

- <https://doi.org/10.1186/s12889-018-5757-2>.
- DOI:10.1371/journal.pone.0159390.
- DOIhttps://doi.org/10.1186/s12879-017-2933-4.
- <https://doi.org/10.3748/wjg.v9.i1.99>.
- <https://doi.org/10.1038/ki.2014.74>

3) Please, add in the strengths and limitations a discussion about this particular design.

**Authors’ response:** Thank you dear editor, we have added one paragraph about the strengths and limitations of nested case-control study, including some references.

**Reviewers’ comments**

**Reviewer #1**

I am still not clear why matching, stratification and adjustment was required for accounting for confounding. I would urge the authors to conduct logistic regression analysis adjusting for all potential confounders at least as a sensitivity analysis including all women recruited in the cohort. I am a great advocate for keeping things simple and it always worries me when complicated statistical analysis is done without any substantial gain.

**Authors’ response**: Thank you again for your time and immense contribution. We acknowledge your comments, which will improve the quality of our manuscript. The main reason for matching is to control confounding like age, and matching in location reduces geographic disparities for such sparse data. Matched analysis (conditional logistic regression) is highly appropriate to manage sparse data problem or sparse data bias.

We also appreciate your suggestion to conduct sensitivity analysis to see whether the conditional logistic regression analysis for nested case-control study could have affected the estimates and the association when full cohort data was analyzed by using the classic (unconditional) logistic regression. According to the sensitivity analysis (supplementary table 1), there is no much discrepancy between the two approaches. The estimates (odds ratios) are nearly the same: for the nested case-control study (conditional logistic regression) AOR=2.97~3.0, 95% CI (1.6, 5.7) while for the cohort study (unconditional logistic regression) AOR=3.26 ~3.3, 95% CI (1.9, 5.7). Since both approaches estimated nearly similar estimates the interpretations are the same; the odds of primary postpartum hemorrhage was 3 times higher for women with inter-pregnancy interval under 24 months as compared to women with inter-pregnancy interval 24-60 months.

END!
